# Graves Opthalmopathy and Psychoendocrinopathies

**DOI:** 10.4103/0974-9233.63079

**Published:** 2010

**Authors:** Asaad A. Ghanem, Mostafa A. Amr, Lamiaa F. Araafa

**Affiliations:** Department of Ophthalmology, Faculty of medicine, Mansoura University, Mansoura, Egypt; 1 Department of Psychiatry, Faculty of medicine, Mansoura University, Mansoura, Egypt; 2 Department of Medical Biochemistry, Faculty of medicine, Mansoura University, Mansoura, Egypt

**Keywords:** Graves Ophthalmopathy, Psychological Changes, Thyroid Specific Antibodies

## Abstract

**Purpose:**

To assess the psychiatric and endocrinological changes in patients with Graves ophthalmopathy (GO).

**Design:**

A prospective, controlled, University Hospital based study

**Subjects and Methods:**

The current study comprised 60 patients diagnosed with GO at Mansoura Ophthalmic Center. Thirty five patients of them with moderate to severe GO formed the study group and twenty five patients with negligible to very mild GO formed the control group in the euthyroid state. The study group was further subdivided based on their predominant clinical signs into a proptosis subgroup and a muscle restriction subgroup. Psychiatric changes were assessed with the Middlesex Hospital Questionnaire (MHQ). Biochemical analyses included serum-free thyroxine and thyroid-stimulating hormone (TSH) concentrations, TSH receptor antibody (TRAb) activity and anti-thyroglobulin particle agglutination (TGPA) and antithyroid microsomal particle agglutination (MCPA).

**Results:**

The proptosis group reported significantly higher scores on anxiety, depression, and phobia than the muscle restriction group (*P*<0.0001). The proptosis and muscle restriction subgroups reported significantly higher scores on all subscales compared to the control group (*P*<0.05). The scale scores of depression and phobia showed a positive correlation with scores of anxiety (*P*<0.0001). The serum TRAb activity showed a significant correlation with anxiety, phobia and hysteria (*P*<0.0001).

**Conclusion:**

The psychiatric aspect of GO should be evaluated during routine follow-up and should be considered when making management decisions. Thyroid specific antibodies may be useful in confirming the diagnosis of GO.

## INTRODUCTION

Graves ophthalmopathy (GO), associated with Graves thyroid disease (GTD), is an incapacitating eye disease, causing disfiguring proptosis, pain, redness, swelling of the eyelids, grittiness of the eyes, diplopia, and sometimes blindness.^1^ Supportive medical therapy is required for approximately 50% of the patients with mild symptoms. In patients with moderately severe active disease management may include surgery.^2^ The outcomes of GO disease and treatment are mostly assessed with biological and physiological measures such as the NO SPECS classification.^3^This classification of eye changes in Graves ophthalmopathy (Werner's classification) includes class 0 indicating no physical signs or symptoms, class 1 indicating only signs, no symptoms (eg, upper lid retraction, stare, and eyelid lag); class 2 indicating soft tissue involvement (symptoms and signs); class 3 indicating proptosis; class 4 indicating extraocular muscle involvement; class 5 indicating corneal involvement, and class 6 indicating visual loss (optic nerve involvement).

While these measures provide important information to clinicians, they often correlate poorly with functional capacity and perceived health as experienced by the patient.^4^ For example, Prummel *et al.*^5^ found a response rate of 50% and 46% respectively to prednisone and radiotherapy measured with the NO-SPECS classification. However, the benefit of both treatments on the subjective judgment of the eye condition by the patient was only modest. Furthermore, the psychological burden of the progressive disfigurement resulting from GO is well recognized.^6^

Bartley *et al*.^7^reported 61% of the patients believed that the appearance of their eyes had not returned to baseline, 51% thought their eyes continued to be abnormal in appearance, and 37% were dissatisfied with the appearance of their eyes after treatment. Overall, the effects of GO on physical and psychological functioning have a significant impact on patients' health related quality of life.^8^This decrease in daily functioning and perception of health in general has been shown to persist many years after diagnosis and treatment.^9^

Health related quality of life assessment is valuable for determining the effects of treatment for GO. Currently, a small number of studies have examined the clinically significant psychological impairment compared to the degree of disfigurement from GO that would adversely affect the treatment regimen including early surgical intervention or perhaps a delay if psychological impairment is severe.^10^

The present study investigated the association between Graves ophthalmopathy and psychoendocrinopathy morbidity in the middle aged population.

## MATERIALS AND METHODS

This was a prospective, controlled, comparative study on subjects with GO who completed the Middlesex Hospital Questionnaire. formulated by Crown and Crisp.^11^ The study was approved by a medical ethics committees and written informed consent was obtained from all subjects prior to enrollment. This study was conducted in accordance with the Declaration of Helsinki.

### Patients

This study was conducted on 60 subjects with GO aged 45-53 years who presented at Mansoura Ophthalmic Center, Mansoura, Egypt. The subjects were divided into two groups; the first group comprised 30 subjects with negligible to mild GO (control group) and the second group comprised 35 subjects with moderate to severe GO (study group). The study group was subdivided into 20 subjects with predominant proptosis (proptosis group) and 15 subjects with predominant muscle restriction (muscle restriction group).

Inclusion criteria were patients with diagnosed GO based on history and clinical examination (in some subjects the diagnosis was confirmed by computed tomography), euthyroid at the time of evaluation based on recent thyroid-stimulating hormone (TSH) levels, sufficient cognitive ability to provide accurate information, no use of antidepressant, anti-anxiety, anti-inflammatory or mood-stabilizing medications immediately prior to or for the duration of the study, and no other unstable eye disease.

Exclusion criteria were patients with ocular disease that mimicked symptoms such as myasthenia gravis, patients with concomitant glaucoma, and patients with no recent clinical eye measurements.

### Case definition

Moderate to severe GO was defined as proptosis greater than 22 mm in either eye and/or significant extraocular movement (EOM) restriction greater than −1.5 in any direction of gaze and in either eye. The study group was further subdivided based on whether their predominant clinical signs consisted of proptosis (proptosis greater than 22 mm and EOM restriction greater than −1.5) or muscle involvement (EOM restriction greater than −1.5 and proptosis less than 22mm). Negligible to mild GO was defined as proptosis of 21 mm or less and EOM restriction of −1 or less in both eyes, no lagophthalmos or no lid or periorbital edema and no diplopia.

Complete ophthalmic examination was performed and an assessment of the severity and activity of GO was performed including: assessment of proptosis using a Hertel exophthalmometer, measurement of eye motility using −4 to +4 scale for ductions, assessment of visual acuity, measurement of intraocular pressure by Goldmann applanation tonometry at primary gaze and upgaze, severity of lagophthalmos, marginal reflex distance measurement, and severity of lid /periorbital oedema. If visual acuity was reduced, the cornea was stained with fluorescein, and examined with the slit-lamp. If the fundus examination normal despite reduced visual acuity, optic nerve function was assessed with visual evoked potential.

### Psychometric testing

All the subjects were first evaluated with a specially designed questionnaire to assess; demographic characteristics of subjects with GO (age, gender, marital status), clinical characteristics that include general factors (fatigue, inappropriate weight gain, palpitations and smoking), ocular (eye lid retraction, proptosis, diplopia, visual field defect, muscle restriction, optic atrophy, prior systemic steroid alone or with radiotherapy and prior orbital /lid/muscle surgery)and psychiatric (family history of psychiatric disorder, past history of psychiatric disorder, past history of psychotropic medication intake).

Subsequently, the psychiatric changes were assessed with the Middlesex Hospital Questionnaire (MHQ) formulated by Crown and Crisp.^11^ The objective of the MHQ is to obtain a score that can be used as a diagnostic and prognostic tool in research. It is short self-rating scale comprised of 48 items with 6 subscales covering 6 groups of psychiatric symptoms: anxiety, phobia, obsessive compulsive symptoms, somatic symptoms, depressive symptoms and hysteria. The response to each item is scored by either 2,1 or zero.

### Thyroid function tests

Serum-free thyroxine (T4) and TSH concentrations were measured with radioimmunoassay. Serum TSH receptor antibody (TRAb) activity was determined by radioreceptor assay. Anti-thyroglobulin particle agglutination (TGPA) and antithyroid microsomal particle agglutination (MCPA) were determined by haemagglutination method.

### Statistical analysis

Data were analyzed using SPSS version 15 (SPSS Inc, Chicago, Ill., USA). The unpaired *t*-test and Chi-square χ*^2^* were used to detect differences in either demographic or clinical characteristics. Analysis of variance (ANOVA) was used to detect significant differences in MHQ scores in both groups. The correlation coefficient was used to test the association between variables. A *P* value less than 0.05 was considered statistically significant.

## RESULTS

Table 1 summarizes the demographic and clinical profiles in subjects classified as euthyroid. Age, residence, gender, marital status and duration of GO were not significantly different between the three groups (*P*=0.54, *P*=0.23, *P*=0.34, *P*=0.56, *P*=0.38 respectively). General, ocular and psychiatric manifestations were not significantly different(*P*=0.31, *P*=0.47 and *P* = 0.41) between the three groups.

**Table 1:** Demographic and clinical characteristics of the Graves Ophthalmopathy subgroups and Control group

Parameters	Control group (C) (n= 25)	Study group (S) (n=35)		Statistical analysis*
			
		Proptosis (P) (n=20)	Muscle restriction (M) (n=15)	
Mean Age± SD (years)	51.31± 3.51	53.42± 4.51	55.37± 1.42	*F*=5.46
				*P*=0.54
Residence:				
Rural	18(72)	11(55)	9(60)	χ^2^=0.004
Urban	7(28)	9(45)	6(40)	*P*=0.23
Gender:				
Female	17(68)	12(60)	9(60)	χ^2^=0.005
Male	8(32)	8(40)	6(40)	*P*=0.34
Marital states:				
Married	13(52)	9(45)	9(60)	*F*=6.45
Single	8(32)	7(35)	4(27)	*P*=0.56
Widowed divorced	4(16)	4(20)	2(13)	
Duration of GO (months)				
0-6	14(56)	12(60)	8(53)	χ^2^=0.006
6-18	11(44)	8(40)	7(47)	*P*=0.38
General:			
Fatigue	14(56)	8(40)	6(40)	*F*=5.67
Inappropriate weight gain	8(32)	4(20)	4(26)	*P*=0.31
Palpitation**	11(44)	7(35)	6(40)	
Smoking	8(32)	4(20)	0	
Ocular				
Eyelid retraction	13(52)	4(40)	8(53)	*F*=4.76
Visual field defect	11(44)	5(25)	4(26)	*P*=0.47
Diplopia	8(32)	3(15)	4(26)	
Optic atrophy	0	2(10)	2(13)	
Prior systemic steroid therapy	14(56)	7(35)	6(40)	
Prior systemic radiotherapy	3(12)	4(20)	3(20)	
Prior steroid and radiotherapy	2(8)	3(15)	3(20)	
Prior orbital / lid / muscle surgery	0	1(5)	1(6)	
Psychiatric:				
Family history of psychiatric disorder	3(12)	2(10)	0	*F*=2.57
Past history of psychiatric disorder	2(8)	0	2(13)	*P*=0.41
Past history of medication intake	6(24)	5(25)	3(20)	

*F*=One Way Analysis of variance test, χ^2^ denotes Chi-square test, *P*≤0.05 was considered statistically significant

** Palpitation = greater than or equal to 110 per minute

Comparison of mean MHQ scale scores in the control and study groups show that anxiety, phobia and depression were significantly higher in the proptosis group than those of the muscle restriction group [Table 2, *P* < 0.0001]. Obsessive compulsive disorder, somatization and hysteria were not significantly different (*P*>0.05). Obessive compulsive disorder, anxiety, phobia, depression and hysteria scores were significantly higher in the proptosis group than those of the control group (*P*<0.0001).

**Table 2:** Results from the Middlesex Hospital Questionnaire of the Graves Ophthalmopathy subgroups and Control group

Parameters	Control group (C) (n= 25)	Study group (S) (n=35)	
		
		Proptosis (P) (n=20)	Muscle restriction (M) (n=15)
Anxiety			
Range	14.5-5.8	19.5-7.7	15.7-5.8
Mean± SD	11.6±2.8	17.5±2.1	12.6±2.7
*P* value		t<0.0001	t_1_<0.0001
			t_2_<0.0001
Phobia			
Range	9.7-3.8	20.7-7.56	14.8-5.5
Mean± SD	6.5±2.9	18.2±4.1	11.6±3.9
*P* value		t<0.0001	t_1_<0.0001
			t_2_<0.0001
Obsession			
Range	11.4-4.7	16.4-5.5	12.8-6.9
Mean± SD	8.5±3.1	12.2±3.7	8.7±3.6
*P* value		t<0.0001	t_1_<0.0001
			t_2_=0.75
Somatization			
Range	12.6-5.9	17.8-6.9	20.8-9.7
Mean± SD	9.3±4.1	14.6±4.5	18.6±3.9
*P* value		t<0.0001	t_1_<0.0001
			t_2_=0.68
Depression			
Range	8.9-3.7	13.8-7.7	14.4-6.7
Mean± SD	5.1±4.1	10.6±3.7	8.5±3.6
*P* value		t<0.0001	t_1_<0.0001
			t_2_<0.0001
Hysteria			
Range	9.7-3.6	14.9-6.7	15.9-6.1
Mean± SD	7.5±4.2	12.6±4.5	8.5±2.9
*P* value		t<0.0001	t_1_<0.0001
			t_2_=0.97

*F* denotes the F statistic for the one way analysis of variance test, χ^2^ denotes Chi-square statistic, *P*≤0.05 was considered statistically significant, t=between C & P, t_1_=between C & M, t_2_=between P&M

Table 3, presents the estimated thyroid function tests in the control and study groups. Serum TRAb activity, MCPA, and TGPA levels were significantly higher in the proptosis group than in the control group [Table 3, *P*<0.0001]. Serum TRAb activity, MCPA ,and TGPA levels were also significantly higher in the muscle restriction group than the control group [Table 3, *P*<0.0001].

**Table 3:** Estimated thyroid function parameters of the Graves Ophthalmopathy subgroups and Control group

Parameters	Control group (C) (n= 25)	Study group (S) (n=35)	
		
		Proptosis (P) (n=20)	Muscle restriction (M) (n=15)
FT4 (pmol/L)			
Range	18.5-3.7	20.3-5.6	20.3-4.8
Mean ± SD	14.2±2.7	16.3±4.5	16.5 3.96
*P* value		t=0.173	t_1_=0. 185
			t_2_=0.163
TSH (MU/L)			
Range	3.5-0.05	4.6-1.8	3.57-1.51
Mean ± SD	1.6±1.2	1.6±2.5	1.69± 2.3
*P* value		t=0.081	t_1_=0. 095
			t_2_=0.761
TRAb (%)			
Range	0.9-0.1	6.8-1.9	7.41-1.51
Mean ± SD	0.8±1.8	4.8±2.3	5.8 ±2.1
*P* value		t<0.0001	t_1_<0. 0001
			t_2_=0.653
MCPA (%)			
Range	0	95.6-34.8	98.7-38.1
Mean ± SD		81.6±1.7	92.5 ± 2.1
*P* value		t<0.0001	t_1_<0. 0001
			t_2_=0.867
TGPA (%)			
Range	0	36.5-18.6	38.2-17.8
Mean ± SD		27.5±2.4	32.4 ±3.4
*P* value		t<0.0001	t_1_<0. 0001
			t_2_=0.0813

t=between C&P, t_1_ =between C&M, t_2_= between P&M

* significant at *P*≤0.05

As shown in Table 4, serum TRAb activity was significantly correlated to anxiety, phobia, and hysteria (*r*=0.365, *P*<0.0001; *r*=0.415, *P* < 0.0001, and *r*=0.517, *P*<0.0001 respectively). There was a positive correlation of depression with phobia (*r*=0.363, *P* < 0.0001). There was a positive correlation of phobia with anxiety (*r*=0.851, *P* < 0.0001).

**Table 4:** Correlation between psychological factors and thyroid-related parameters

	Anxiety	Phobia	Obsession	Somatization	Depression	Hysteria	Serum TRAb	Serum FT4
Anxiety	−	0.851*	0.179	0.173	0.165	0.018	0.365*	-0.056
Phobia		−	0.225	0.185	0.363*	0.031	0.415*	-0.076
Obsession			−	0.197	0.153	0.080	0.083	-0.081
Somatization				−	0.145	0.022	0.145	-0.042
Depression					−	0.073	0.191	-0.068
Hysteria						−	0.517*	-0.012
Serum TRAb							−	-0.088
Serum FT4								−

* Significant at P≤0.05, TRAb denotes thyroid-stimulating hormone receptor antibody, FT4 denotes serum-free thyroxine

## DISCUSSION

Graves ophthalmopathy has been the subject of numerous published reports, yet information on the epidemiologic, clinical, and psychological characteristics in an incidence cohort have not been reported until recently.^12^ It is known that psychological abnormalities exist in patients with Graves disease, and such abnormalities may be due to hyperthroidism. A temporal assocation between the onset of hyperthyroidism and GO exists. However 4-18% of patients with Graves disease develop thyroid dysfunction after the onset of GO. Hence laboratory workup of thyroid function is warranted in euthyroid patients.^13^ In the present study the subjects were euthyroid when they completed the MHQ questionnaires. We found disfigurement and diplopia had a significant impact on the patient's health related quality of life. Using MHQ as a screening test for psychiatric symptoms in subjects with GO, we found that those who have noticeable proptosis and/or who have functionally limiting double vision have significant symptoms of anxiety, depression and phobia compared with subjects who have mild or negligible symptoms.

The outcomes of the present study concur with Hall^14^who reported that between 30% and 40% of patients with GO presented with conspicuous complaints of anxiety, nervousness, apprehension, dread, depression, restlessness, diminished concentration, forced thinking, emotional lability, and hyperkinesia. Jefferson and Marshall^15^ report that nervousness exhibited by hyperthyroid patients is characterized by restlessness and shortness of attention span which differs from patients with a primary anxiety neurosis. Trepacz *et al*.^16^reported a high prevalence of general anxiety disorder in a series of patients with untreated Graves disease. Emanuele *et al.*^17^ presented four cases of coexisting agoraphobia and hyperthyroidism, where the patients reported a fear of crowded or confined spaces, difficulty traveling away from home or places of safety and the development of panic attacks. The results of our study demonstrated high levels of anxiety, phobia, and depression in subjects with GO. Furthermore,the degree of depression and anxiety was related to both visual changes and disfigurement of the eye. These results are consistent with Kahaly *et al.*,^18^ and Coulter *et al.*^19^

When subjects with GO were divided into those with predominantly disfiguring signs (proptosis-- the forward displacement of the eyeball) and those with predominantly functional signs (muscle restriction), we found that it was the disfiguring aspect of the disease that accounted for much of the psychiatric symptoms. It was apparent that the disfigurement caused much of the psychiatric disturbance. Farid *et al.*^10^ proposed that this progressive disfigurement be recognized as an indication for orbital decompression surgery. In such cases, major complications such as visual loss are rare and diplopia may occur but is treatable with subsequent strabismus surgery. In some cases, corrective surgery may be considered and a lower threshold for surgical intervention in patients with significant Graves ophthalmopathy-related psychiatric disturbances might have a significant benefit on quality of life.

Seo *et al*.^20^reported the association of severity of eye disease in GO with neuropsychiatric disease. There have been reported cases of encephalopathy as well as psychiatric disease that may be related to the presence of the thyroid related autoantibodies. Eckstein *et al.*^13^ reported that positive TRAb levels confirmed the diagnosis of GO in 69% of the euthyroid and hypothyroid patients. The TRAb and thyroid peroxidase antibodies (TPOAb) titres combined confirmed the diagnosis of thyroid associated ophthalmopathy in 75% of subjects. Matuzas *et al*.^21^ assessed 65 self-referred patients with panic attacks for thyroid abnormalities. Twenty-five percent of the women from 30 to 40 years old, had positive antithyroid antibodies compared to 5% to 13.8% of women of similar age in the general population.^21^ Matuzas *et al.*^21^ suggested that the prevalence of thyroid antibody titers was elevated in patients with panic attacks. Nemeroff *et al*.^22^ had previously noted that 8 of 53 patients (15%) suffering from depression had elevated thyroid microsomal antibody titers. However, Paschke *et al.*^23^ studied 15 female subjects with Graves disease and noted that their psychological parameters showed considerable change as their thyroid status improved.

In the present study, anxiety, phobia and depression scores correlated with serum TRAb activity, suggesting that the impact of psychological changes lead to aggravation of the autoimmune abnormality and an interaction between endocrinopathies and psychoneuropathies.

Graves ophthalmopathy is a debilitating disease associated with significant psychological and endocrinal burden, especially when disfiguring signs are predominant. The psychiatric aspect of the disease should be evaluated in routine follow-up and should be taken into consideration when making management decisions which may be useful in improving the prognosis of Graves disease. Also, thyroid antibodies are specific indicators for thyroid autoimmunity ,which may be useful in confirming the diagnosis of the thyroid associated ophthalmopathy.
